# Three Groups in the 28 Joints for Rheumatoid Arthritis Synovitis – Analysis Using More than 17,000 Assessments in the KURAMA Database

**DOI:** 10.1371/journal.pone.0059341

**Published:** 2013-03-12

**Authors:** Chikashi Terao, Motomu Hashimoto, Keiichi Yamamoto, Kosaku Murakami, Koichiro Ohmura, Ran Nakashima, Noriyuki Yamakawa, Hajime Yoshifuji, Naoichiro Yukawa, Daisuke Kawabata, Takashi Usui, Hiroyuki Yoshitomi, Moritoshi Furu, Ryo Yamada, Fumihiko Matsuda, Hiromu Ito, Takao Fujii, Tsuneyo Mimori

**Affiliations:** 1 Center for Genomic Medicine, Kyoto University Graduate School of Medicine, Kyoto, Japan; 2 Department of Rheumatology and Clinical Immunology, Kyoto University Graduate School of Medicine, Kyoto, Japan; 3 Department of the Control for Rheumatic Diseases, Kyoto University Graduate School of Medicine, Kyoto, Japan; 4 Department of Clinical Trial Design and Management, Translational Research Center, Kyoto University Hospital, Kyoto, Japan; 5 Department of Orthopaedic Surgery, Kyoto University Graduate School of Medicine, Kyoto, Japan; 6 Unit of Statistical Genetics Center for Genomic Medicine, Kyoto University Graduate School of Medicine, Kyoto, Japan; 7 Institut National de la Sante et de la Recherche Medicale (INSERM) Unite U852, Kyoto University Graduate School of Medicine, Kyoto, Japan; 8 CREST Program, Japan Science and Technology Agency, Kawaguchi, Saitama, Japan; Auburn University, United States of America

## Abstract

Rheumatoid arthritis (RA) is a joint-destructive autoimmune disease. Three composite indices evaluating the same 28 joints are commonly used for the evaluation of RA activity. However, the relationship between, and the frequency of, the joint involvements are still not fully understood. Here, we obtained and analyzed 17,311 assessments for 28 joints in 1,314 patients with RA from 2005 to 2011 from electronic clinical chart templates stored in the KURAMA (Kyoto University Rheumatoid Arthritis Management Alliance) database. Affected rates for swelling and tenderness were assessed for each of the 28 joints and compared between two different sets of RA patients. Correlations of joint symptoms were analyzed for swellings and tenderness using kappa coefficient and eigen vectors by principal component analysis. As a result, we found that joint affected rates greatly varied from joint to joint both for tenderness and swelling for the two sets. Right wrist joint is the most affected joint of the 28 joints. Tenderness and swellings are well correlated in the same joints except for the shoulder joints. Patients with RA tended to demonstrate right-dominant joint involvement and joint destruction. We also found that RA synovitis could be classified into three categories of joints in the correlation analyses: large joints with wrist joints, PIP joints, and MCP joints. Clustering analysis based on distribution of synovitis revealed that patients with RA could be classified into six subgroups. We confirmed the symmetric joint involvement in RA. Our results suggested that RA synovitis can be classified into subgroups and that several different mechanisms may underlie the pathophysiology in RA synovitis.

## Introduction

Rheumatoid arthritis (RA) is the most frequent inflammatory arthritis worldwide affecting 0.5 to 1% of the population [1]. As RA is a bone-destructive disease and functional impairment caused by joint damage is well correlated with swelling and tenderness of joints [2]–[3], the evaluation of joints in patients with RA is very important to assess disease activity and predict the risk of future joint deformity. ACR core set [4] and DAS (disease activity score) [5]–[6] were developed for evaluation of disease activity in RA. Recently, the three composite indices, namely, DAS28 [5], simplified disease activity index (SDAI) [7] and clinical disease activity index (CDAI) [8] are frequently used for disease activity evaluation among rheumatologists. All of the three indices are shown to be well correlated with future joint destruction [7], [9]. These three methods include the same 28 joints for evaluation of disease activity, namely, bilateral wrist, 1^st^ to 5^th^ metacarpal (MCP) joints and proximal interphalangeal (PIP) joints, elbow, shoulder, and knee joints. Though RA is known to show symmetric joint symptoms [10], the frequency of bilateral joint symptoms and the correlations between each joint symptom are not fully analyzed by using large numbers of joint assessments. There are several reports of successful prediction of joint damage using a reduced number of joints for evaluation by ultrasonography [11]–[12]. These reports raise the possibility that some of the 28 joints are less frequently involved, and are less informative for disease activity. Analyses for characterization of joint symptoms would uncover correlations of unexpected joint symptoms and distribution of synovitis in RA.

Here, we analyzed the distribution of affected joints in the 28 joints in patients with RA using more than 17,000 joint assessments from 1,314 patients with RA and showed that synovitis in RA patients can be classified into three groups. We also showed that affected rates of the 28 joints greatly vary in RA patients, and that RA patients could be classified into subgroups based on the distribution of joint synovitis.

## Results

### Frequency order of joints involvement

**Figure 1:**
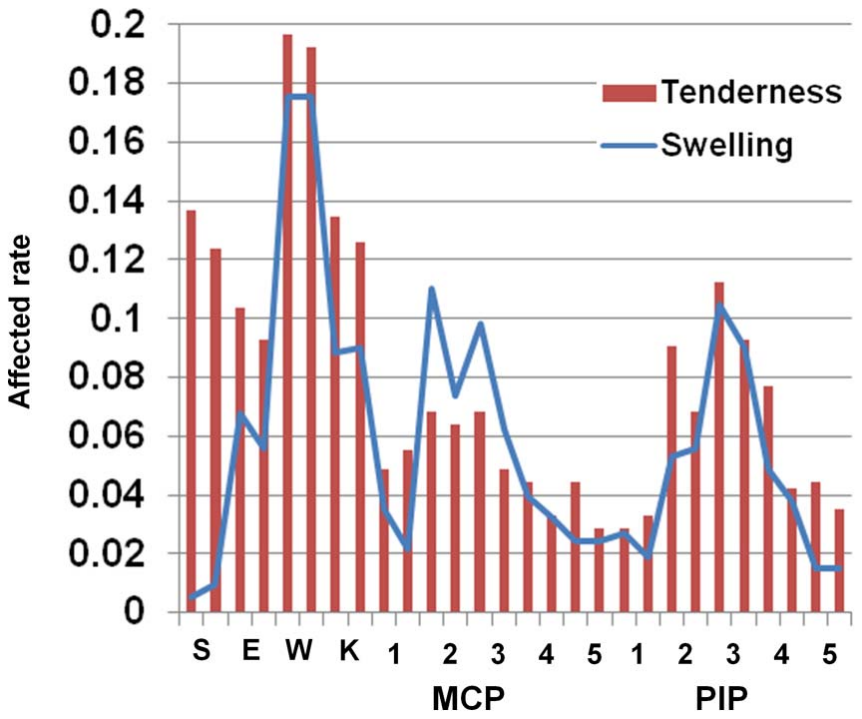
Affected rate of joint symptoms. Affected rate of joint symptoms. Each joint is arranged in the order of right and left. S:shoulder, E:elbow, W:wrist, K:knee.

We recruited 17,311 assessments for the 28 joints in 1,314 patients with RA from 2005 to 2011. A summary of the registered patients is listed in Table 1. The distribution of the number of patients with RA in each year and the number of joint assessments for each patient are shown in Figure S1. We analyzed how often each of the 28 joints was tender or swollen in patients with RA in 2011. From the analysis of 735 patients, we found that the frequency of joint swelling and tenderness in the 28 joints is widely different from joint to joint (Figure 1 and Table S1). The wrist joints were the most frequently affected joints for swelling and tenderness. The frequency of the right wrist joint being affected was more than four times as high as the least frequently affected joint. Many of the joints showed right-dominant tenderness (eleven of fourteen joints, p = 0.057, binomial test), indicating mostly right-handedness. We found strong correlations for the affected rates of each joint between swellings and tenderness except for shoulder joints (Spearman's rank-sum coefficient, rho = 0.70 and p = 3.8×10^−5^, Figure 1, Table S1). Shoulder joints showed much higher frequencies of tenderness than those of swellings.

**Table 1:** Summary of the KURAMA database.

	The KURAMA database
RA patients	1314
Age (mean±SD)	60.2±15.1
female ratio	81.70%
disease duration (years)	12.2±9.8
Stage*	2.75±1.17
Class*	1.87±0.69

* Stage and Class represent Steinbrocker's stage and class, respectively.

SD: standard deviation.

Next, we tried to replicate the order of affected frequencies of the 28 joints and the correlation between tenderness and swellings in different RA patients. We obtained 579 patients whose joints data were not available for 2011, indicating we analyzed independent RA patients. We found that the order of the affected joint frequencies were well correlated for both swelling and tenderness among different sets of RA patients (Spearman's rank-sum coefficient, rho:0.815 and 0.904, p = 1.3×10^−7^ and p = 4.6×10^−11^ for swelling and tenderness, respectively, Figure S2). We also confirmed that rates of tenderness were well correlated with those of swellings in the 28 joints in the 579 patients (rho:0.604). These results indicate that some of the 28 joints are more likely to develop arthritis than the others in RA patients. The swelling and tenderness correlate with each other except for shoulder joints.

Whether the right-dominant involvement of joints in patients with RA is associated with joint destruction was analyzed. Joint destruction in the hand was evaluated for 246 patients with RA by modified Sharp score [13]. The six elements of the scores were separately analyzed, namely erosion of PIP, MCP, and wrist joints (we defined as joints other than MCP and PIP in hand) and narrowing of PIP, MCP, and wrist joints. We found that five out of six elements showed right-dominant destruction. In particular, narrowing and erosion of MCP joints showed a statistically significant right-dominance in binomial test (p< = 0.0050, Table S2).

### Three groups of 28 joints in RA synovitis

**Figure 2:**
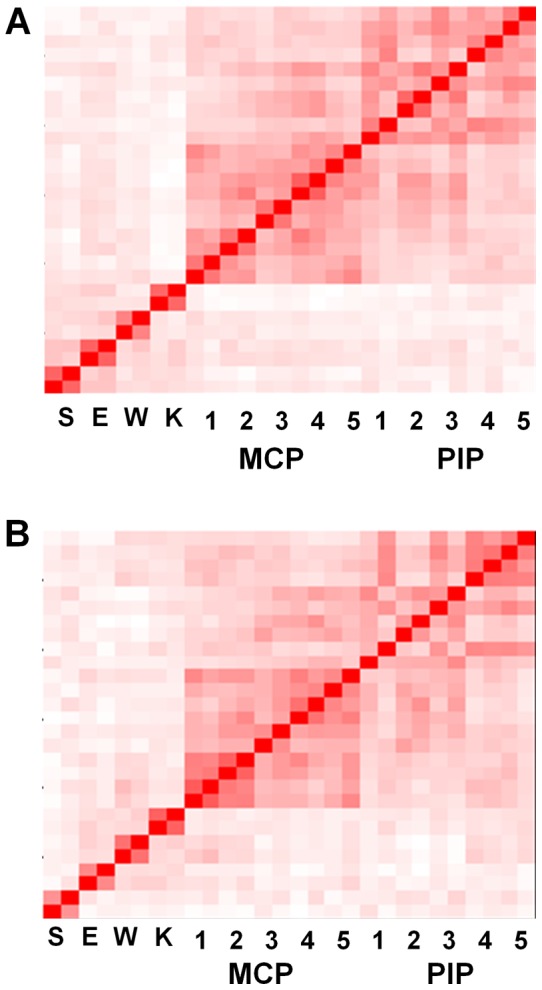
Correlations between the 28 joint symptoms. Brightness of the red color corresponds to the strength of correlations between joint tenderness (A) or swellings (B), using the Kappa coefficient. Each joint is arranged in the order of right and left. The joint order in the y axis is the same as the x axis. The result is a representative of five analyses based on resampled assessments. S:shoulder, E:elbow, W:wrist, K:knee.

Next we analyzed correlations of joint symptoms between the 28 joints. We randomly picked up one assessment from each of the 1,314 patients to maximize the power. When the correlation of tenderness of the 28 joints was analyzed with kappa coefficient, we confirmed that each joint showed a symmetric involvement (Figure 2A). The results also showed that the tenderness of large joints and wrist joints are not correlated with the tenderness of PIP and MCP joints. We found that the tenderness of MCP joints was especially well correlated with each other and that PIP joints tenderness was well correlated with each other. The correlation of swelling in the 28 joints showed the same tendency as that of tenderness, namely, symmetric joint involvement, correlations between large joints and wrist joints, and no strong correlations between wrist joints and other small joints (Figure 2B).

**Figure 3:**
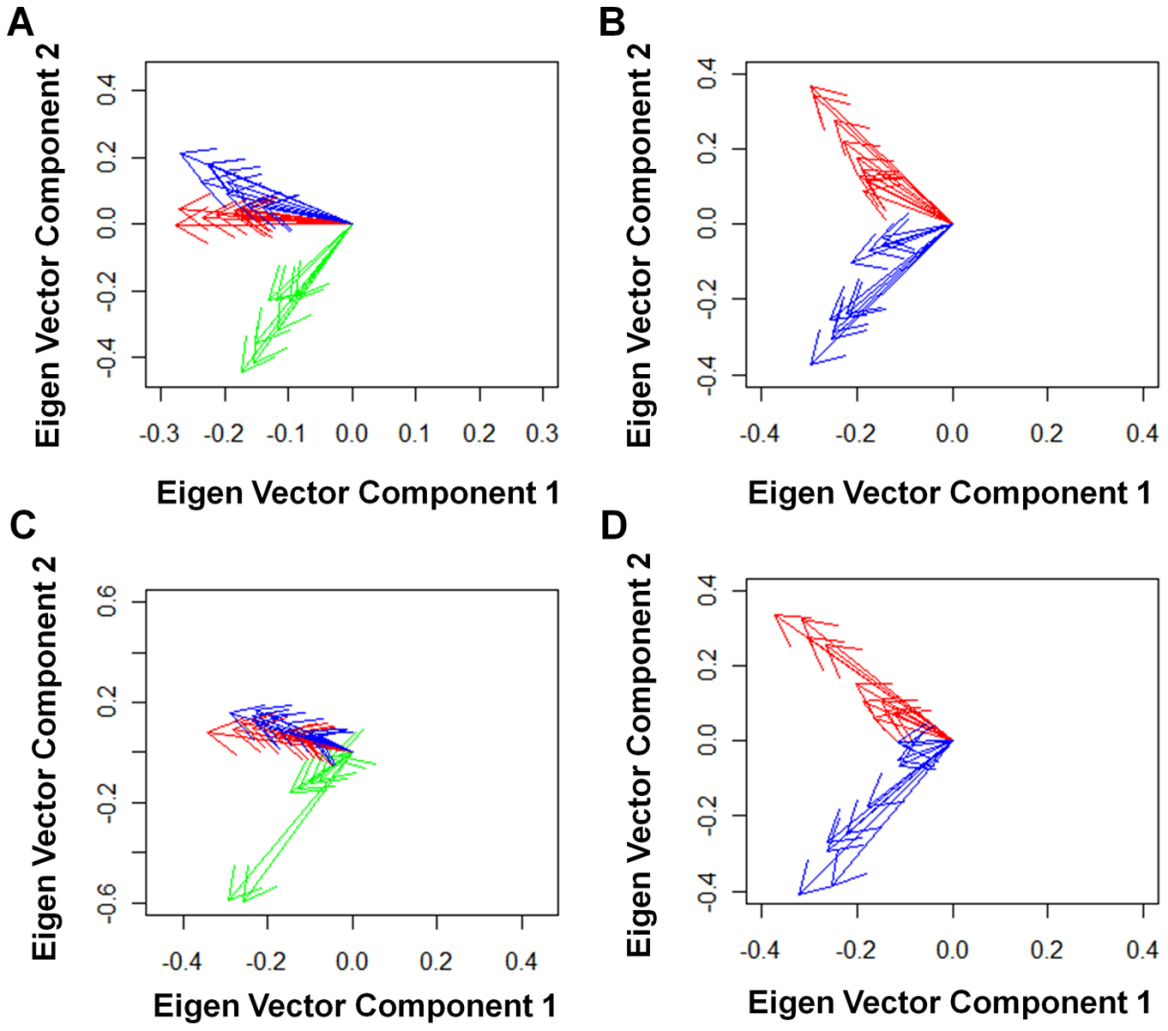
Relationship of the 28-joint involvement. The 1^st^ and 2^nd^ components of eigen vectors of the joint symptoms are plotted, using principal component analysis of the 28 joint involvement for tenderness (A) and swelling (C) or using that of the 20 joint involvement other than large and wrist joints for tenderness (B) and swelling (D). The results are representatives of five analyses based on resampled assessments. Green: large and wrist joints. Red: MCP joints. Blue: PIP joints.

Next we used eigen vectors of principal component analysis to assess the correlations of the 28 joints involvement. When we analyzed correlations of tenderness, eigen vectors revealed that PIP and MCP joints can be clearly distinguished from large joints and wrist joints (Figure 3A). PIP joints and MCP joints turned out to make independent groups after excluding large joints and wrist joints (Figure 3B). These three groups of affected joints were found both for tenderness and swelling (Figure 3C and 3D). We confirmed these three correlation groups in four independent resampling analyses by randomly picking up one assessment from each of the 1,314 patients four times (data not shown). The three groups were observed in the two independent sets of RA patients which were used in the analysis of joints involvement frequency (Figure S3). In addition, no significant difference was observed in the relationship of the three groups of joint involvement when we divided the 1,314 patients into two groups according to the patients' caring physicians (Figure S4). We confirmed the three groups by resampling four times for each analysis (data not shown). These results indicate that these three groups were not due to specific patients, examiners, or time of evaluation.

Taken together, the correlation analyses using kappa coefficient and eigen vectors in principal component analysis indicated that there are three correlated groups of joints in RA synovitis, namely, large joints with wrist joints (which we express as “large and wrist joints”), PIP joints, and MCP joints.

### Subgroups of patients with RA

**Figure 4:**
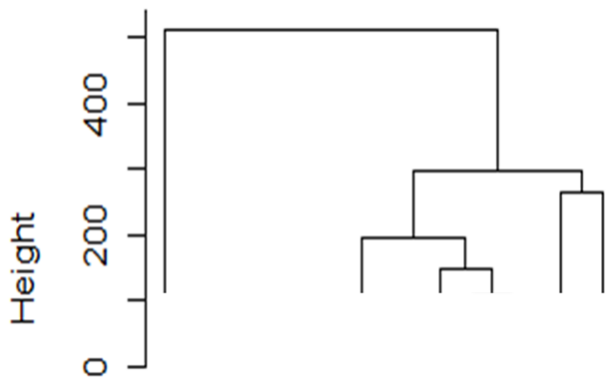
Six subgroups of evaluations of the 28 joints in RA. Results of clustering analysis with Ward method using randomly obtained 5,383 evaluations of the 28 joints in 1,314 patients were plotted.

We performed a clustering analysis of 5,383 evaluations of 28 joints from 1,314 patients with RA. Six subgroups of evaluations of 28 joints were observed (Figure 4). Each of the subgroups was characterized by 1) no synovitis (34.6%), 2) mild activity with dominant involvement of large and wrist joints (17.4%), 3) dominant involvement of MCP joints (18.3%), 4) dominant involvement of PIP joints (9.3%), 5) active synovitis (4.1%), and 6) moderate activity with dominant involvement of large and wrist joints (16.4%) (Table S3). Whether patients with RA are classified into the same subgroups was analyzed. There were 998 patients with four or five evaluations, and of these, 734 were categorized into the regular groups across different evaluations, indicating that the patterns of synovitis in the same patients were stable. Analysis of joint destruction in each subgroup revealed that the sixth subgroup demonstrated dominant destruction of large and wrist joints compared with MCP and PIP joints (p< = 2.8×10^−5^, Figure S5 and Figure S6).

## Discussion

Since RA is a joint destructive autoimmune arthritis and joint damage occurs rapidly in the early stages of the disease course [14], the development of a quantitative scale which assesses disease activity and predicts joint damage is very important. After DAS and ACR core sets were introduced, DAS28, SDAI, and CDAI were developed to evaluate disease activity and easily calculate the disease activity score in patients with RA. All three indices were shown to be well correlated with future joint destruction and they share the same 28 joints for evaluation. Joint symptoms especially joint swelling is known to correlate with future joint damage [3]. While these indices were developed for use in clinical trials such as responsiveness to treatment, they are used by rheumatologists in daily clinical practice and they are reported to coincide very well among different examiners [9]. Characterizing the relative affected frequency of each joint and analysis of correlation between joint symptoms are important to analyze the basic mechanisms of synovitis and to efficiently select the joints to predict future joint destruction. However, there is no detailed analysis to address the correlations between the 28-joint symptoms.

In the current study, we characterized the 28-joint symptoms using large numbers of joint assessments. While we reported the affected rates of each joint in the 28 joints for tenderness and swelling of RA patients registered in the KURAMA database in 2011 as a representative (Table S1), these rates should not be generalized considering large effects of treatment especially biologics agents on joint symptoms. Thus, we focused on relative frequencies of joint involvement for the 28 joints. The affected frequency pattern was compared between the two sets of RA patients, and there were no apparent differences between the two sets for both tenderness and swelling. We also showed that joint symptoms in RA could be classified into three groups both for tenderness and swelling. Our analysis also demonstrated that patients with RA can be regularly classified into six subgroups based on patterns of joint symptoms. These results suggest that regular RA joint involvement pattern, including relative frequency and groups of joints, is largely maintained in RA patients. In addition, we confirmed that these patterns of joint involvement were not attributed to evaluators and fractions of RA patients.

It is interesting that the affected frequencies greatly varied from joint to joint, and the rate of the most highly affected joint was more than four times as high as the least-affected joint. The affected frequencies indicated that wrist joints were the most frequently affected. It should be noted that surface area may have influenced the sensitivity of detecting synovitis in physical exams when different joints were compared. The relatively high frequency of tenderness and swelling in large and wrist joints compared with MCP and PIP joints can be explained by this difference in surface area. However, surface area cannot fully explain the highest frequency of wrist involvement and different frequencies within the MCP or PIP joints. A dominant involvement of right joints seemed to indicate a majority of the study population being right-handed in spite of the small difference of affected rates between bilateral joints. We also demonstrated that the right dominant involvement was also true for joint destruction. We could not compare the joint involvement and joint destruction between right-handed patients and left-handed patients due to a lack of information regarding handedness of patients.

Correlation analysis confirmed the well-known symmetric joint involvement in patients with RA. Strong correlations of tenderness and swelling in the same joints except for shoulder joints may indicate low sensitivity of shoulder swelling in the physical exams and common mechanisms of swelling and tenderness. It is striking that joint symptoms can be classified into three groups based on correlation analysis and principal component analysis. The association observed between the symptoms in the wrist joints and the large joints is worth noting, since wrist joints are regarded as small joints according to ACR/EULAR criteria set in 2010. As wrist joints are much closer to other small joints than large joints, the relationship between wrist joints and large joints cannot be explained by the distance of joints. The distance of joints cannot explain the two different groups of MCP and PIP joints either. While symptoms of large and wrist joints are not related with those of MCP and PIP joints, they were not very strongly correlated with each other, compared with correlations among PIP joints or MCP joints. This may indicate that there are no common strong factors which predispose large and wrist joints to swelling and tenderness in patients with RA.

We also showed that patients with RA can be divided into six subgroups based on these three groups of joint involvement. More than 70% of patients are classified into regular subgroups, indicating that the pattern of synovitis in a patient with RA is stable. When patients who were regularly classified into the first subgroup of patients characterized by no synovitis were removed, more than 60% of patients were still classified into regular subgroups (data not shown), indicating that the stable patterns were observed regardless of activity of RA. As joint destruction was influenced by disease duration, disease activity, and treatment, we analyzed the relative distribution of joint destruction between the three joint groups in a patient with RA. We found that the sixth subgroup of patients, characterized by moderate activity with dominant involvement of large and wrist joints, demonstrated dominant destruction of wrist joints. This suggests that classifying patients with RA into appropriate subgroups would lead to prediction of patterns of joint destruction.

There are reports that evaluating fraction of joints by ultrasonography is a good way to predict future joint damage [11]–[12]. One study reported that 5 of the 28 joints with MTP2 and MTP5 joints, namely, wrist, MCP2, MCP3, PIP2, and PIP3 joints, are enough for ultrasonography evaluation [12]. Their data seems to be consistent with our results as they selected at least two joints from three different groups into which the 28-joint symptoms were classified. As ultrasonography usually surpasses physical examination in terms of the sensitivity to detect synovitis, it is interesting to analyze whether the assessments of synovitis using ultrasonography show the same pattern of synovitis over the 28 joints in RA.

Our results indicate that RA does not develop synovitis in the 28 joints with the same frequency and that the affected rate of each joint greatly varies from joint to joint. These different distributions of joint synovitis would lead to different distribution of joint destruction. Based on our results, the 28 joints can be categorized into three groups, and it is possible that some fractions of the 28 joints are less informative to assess disease activity than others. It would be interesting to develop a novel simplified joint core set, and analyze the correlation between joint damage and activity score based on this. It would be also interesting to characterize each of RA subsets in more detail.

## Materials and Methods

### Ethics Statement

Written informed consent to enroll in the database described below was obtained from most of the patients, but for some patients the information regarding the construction of this database was disclosed instead of obtaining written informed consent. Participants who were informed regarding the construction of the database (instead of obtaining written informed consent) were allowed to withdraw from the study if desired.

All data were de-identified and analyzed anonymously. This study was designed in accordance with the Helsinki Declaration. This study including the consent procedure was approved by the ethics committee of Kyoto University Graduate School and Faculty of Medicine.

### The KURAMA database

The KURAMA (Kyoto University Rheumatoid Arthritis Management Alliance) database was established in 2011 at Kyoto University to store detailed clinical information and specimens from patients with arthritis and arthropathy. The alliance is composed of rheumatic disease-associated departments in Kyoto University Hospital as well as its allied, integrating previous database and specimen collections in each department and allied. A template for electronic clinical charts developed at Kyoto University Hospital in 2004 to evaluate joint involvements in RA patients was used to obtain joint assessments. Rheumatologists evaluated swelling and tenderness of the 28 joints in patients with RA on each visit and filled in the template. The synovitis information of the 28 joints and data for C-reactive protein and erythrocyte sedimentation rate were extracted from electronic clinical charts [15] and stored in the KURAMA database.

### Patients and data of joint assessment

A total of 17,311 joint assessments from 1,314 patients with RA from 2005 to 2011 were obtained in a retrospective manner from the KURAMA database. All of the patients fulfilled ACR revised criteria for RA in 1987 [10] or ACR and EULAR classification criteria for RA in 2010 [16]–[17].

### Analysis of affected frequencies in the 28 joints

RA patients were subdivided depending on whether their data were available in 2011 or not, and the affected frequency in each of the 28 joints was calculated. We compared the order of the affected frequency in the 28 joints between the two patient sets with Spearman's rank-sum coefficient. We separately analyzed the affected rates of joints for swelling and tenderness. When multiple joint assessments in different visits were available in the same patient with RA, we randomly selected one of the assessments as representative in the patient. We compared frequencies between tenderness and swellings for the 28 joints with Spearman's rank-sum coefficient.

### Clustering of patients with RA

Clustering analyses were performed by Ward method, using randomly-selected 5,383 evaluations of the 28 joints from 1,314 patients with RA. These evaluations did not contain more than six assessments from each patient to avoid excess influence of particular patients. Affected rates were calculated for the three groups of joints (namely PIP joints, MCP joints and large and wrist joints) in this clustering analysis. For example, when a patient showed tenderness and swelling for all PIP joints, the affected rate of PIP joints in the patient is 2. When a patient showed tenderness for four MCP joints, the affected rate of MCP joints is 0.4.

RA patients were regarded as belonging to a particular group when more than 60% of evaluations belonging to the same patients with four or five evaluations were classified into the same group.

### Analysis between RA subgroups and joint destruction

Joint destruction of hand joints in 246 patients with RA was evaluated by modified Sharp score by a trained rheumatologist who was not informed of the patients' characteristics (KM). Joint destruction rates were defined for the three groups of joints as a sum of scores divided by the full score in the joints group. For example, when a patient shows 50 as a sum of scores in the large and wrist group, the patient's joint destruction rate for the group is 0.463 (50/108).

### Correlation of the 28 joints and statistical analysis

Correlations of joint symptoms among the 28 joints were estimated separately for tenderness and swelling. We randomly obtained one assessment of the 28 joints in each patient as a representative of the patient's joint assessments for maximization of the power. Kappa coefficient was used to analyze coincidence of joint symptoms in each pair of the 28 joints. Eigen vectors obtained in principal component analysis were used to analyze the deviation of joint symptoms. We resampled joint assessments for each patient and created four other sets of joint assessments. The same correlation analyses were performed using the four resampled assessments to confirm the correlation shown in the first assessment set. Right dominance of the synovitis and joint destruction was analyzed by binomial test. Dominant destruction of joints was evaluated by paired-t test. Statistical analysis was performed by R software or SPSS (ver18).

## Supporting Information

Figure S1 **Distribution of joint evaluation counts and patients across different years.** A) Distribution of number of RA patients according to numbers of 28-joint assessments. B) Distribution of number of patients with RA whose joint assessment data were available from 2005 to 2011 in the KURAMA database.
(TIF)

Figure S2 **Good correlations between joint involvement rates in different sets of RA patients.** Rates of joint involvement for A) swelling and B) tenderness were compared between the two different sets of RA patients. X and Y axes represent rates in the first set of RA patients in 2011 and those in the second set in 2005 to 2010, respectively.
(TIF)

Figure S3 **Three groups of joints regardless of different sets of RA patients.** Analysis using one of four resampled assessments in one of the two sets of RA patients is shown as a representative. The 1^st^ and 2^nd^ components of eigen vectors of the joint symptoms are plotted, using principal component analysis of the 28 joint involvement for tenderness (A) and swelling (C) or using that of the 20 joint involvement other than large and wrist joints for tenderness (B) and swelling (D). Green: large and wrist joints. Red: MCP joints. Blue: PIP joints.
(TIF)

Figure S4 **Three groups of joints regardless of different evaluators.** Analysis using one of five resampled assessments by one of the two groups of medical doctors is shown as a representative. The 1^st^ and 2^nd^ components of eigen vectors of the joint symptoms are plotted, using principal component analysis of the 28 joint involvement for tenderness (A) and swelling (C) or using that of the 20 joint involvement other than large and wrist joints for tenderness (B) and swelling (D). Green: large and wrist joints. Red: MCP joints. Blue: PIP joints.
(TIF)

Figure S5 **Dominant destruction of large and wrist joints in the sixth subgroup of patients with RA.** Box plots indicating the joint destruction rates in the three joint groups in subjects belonging to the sixth subgroup.
(TIF)

Figure S6 **Destruction of large and wrist joints among the six subgroups of RA.** Differences in destruction rates were plotted for each subject in the six subgroups. The difference was defined as: A) destruction rate of group of large and wrist joints – destruction rate of MCP joints and B) destruction rate of group of large and wrist joints – destruction rate of PIP joints.
(TIF)

Table S1 **Rate of joint involvement for 28 joints in RA.**
(DOC)

Table S2 **Right-dominant joint destruction in RA.** Patients who showed unilateral higher or lower scores in each element were analyzed.
(DOC)

Table S3 **Mean affected rates of the three joint groups in the six subgroups of patients with RA.**
(DOC)
