# Supplementary figures and images for: Three Groups in the 28 Joints for Rheumatoid Arthritis Synovitis – Analysis Using More than 17,000 Assessments in the KURAMA Database
Source: PLoS One. 2013 Mar 12;8(3):e59341. doi: 10.1371/journal.pone.0059341 (PMC3595245; doi:10.1371/journal.pone.0059341)

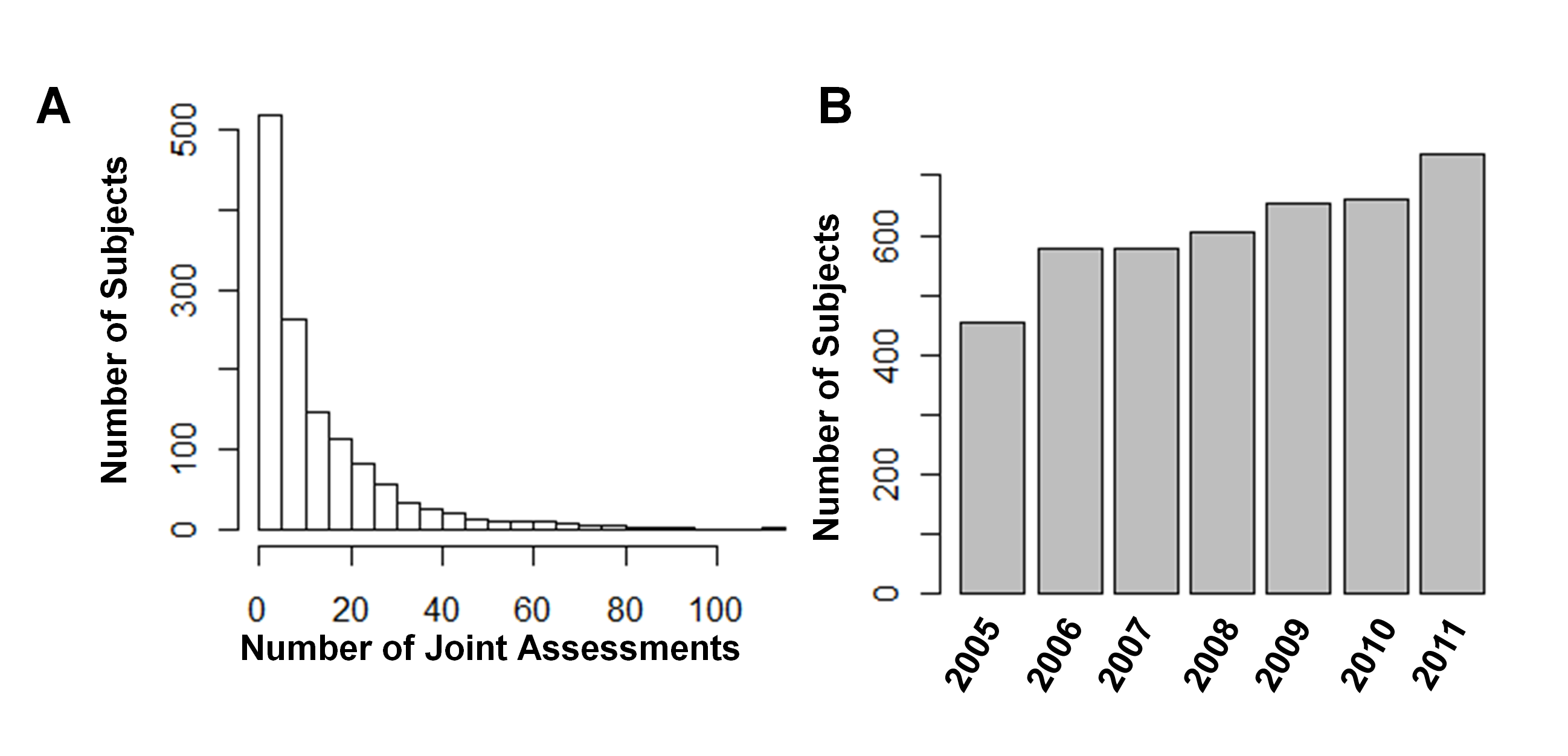

Supplement: Figure S1 — Distribution of joint evaluation counts and patients across different years. A) Distribution of number of RA patients according to numbers of 28-joint assessments. B) Distribution of number of patients with RA whose joint assessment data were available from 2005 to 2011 in the KURAMA database. (TIF) [file pone.0059341.s001.tif]

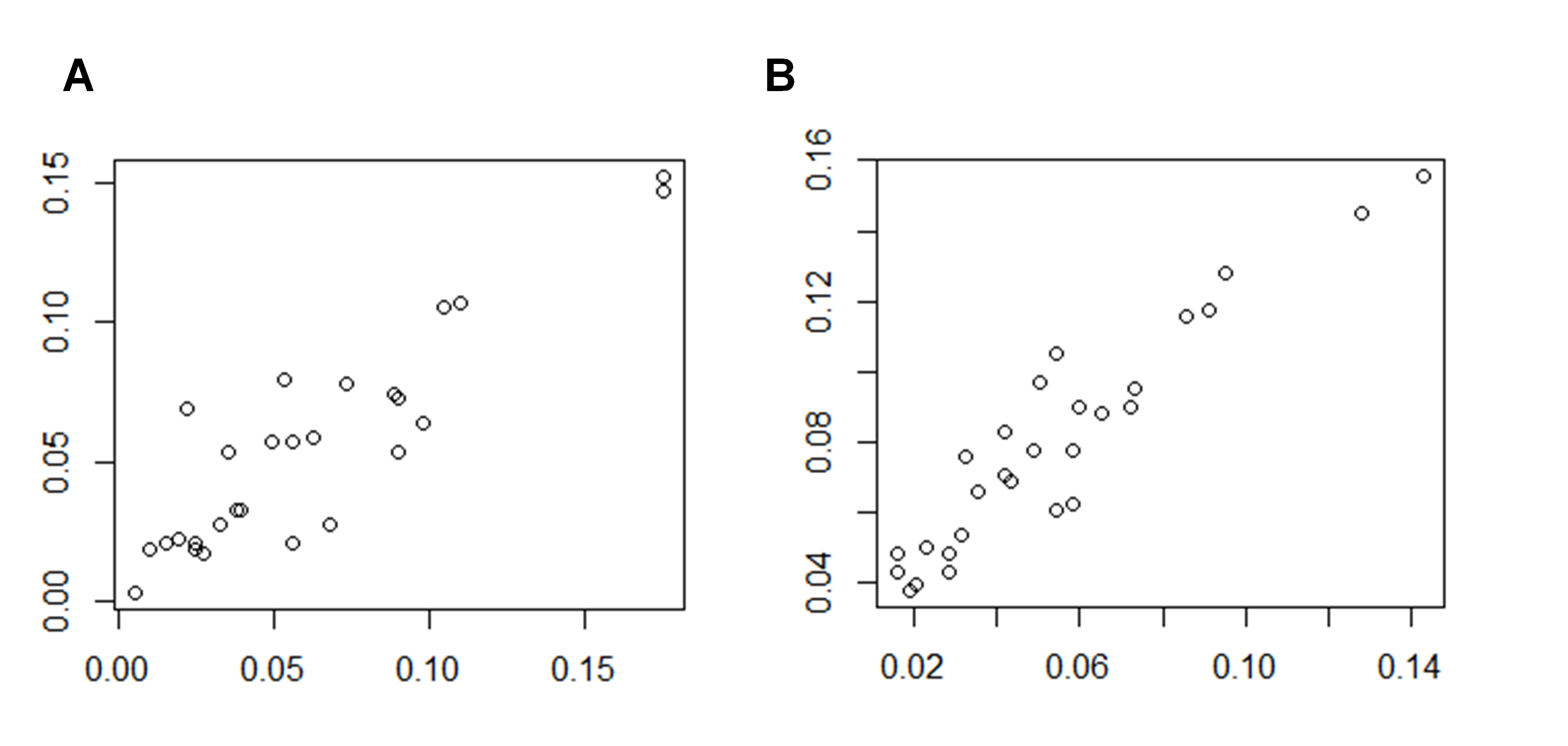

Supplement: Figure S2 — Good correlations between joint involvement rates in different sets of RA patients. Rates of joint involvement for A) swelling and B) tenderness were compared between the two different sets of RA patients. X and Y axes represent rates in the first set of RA patients in 2011 and those in the second set in 2005 to 2010, respectively. (TIF) [file pone.0059341.s002.tif]

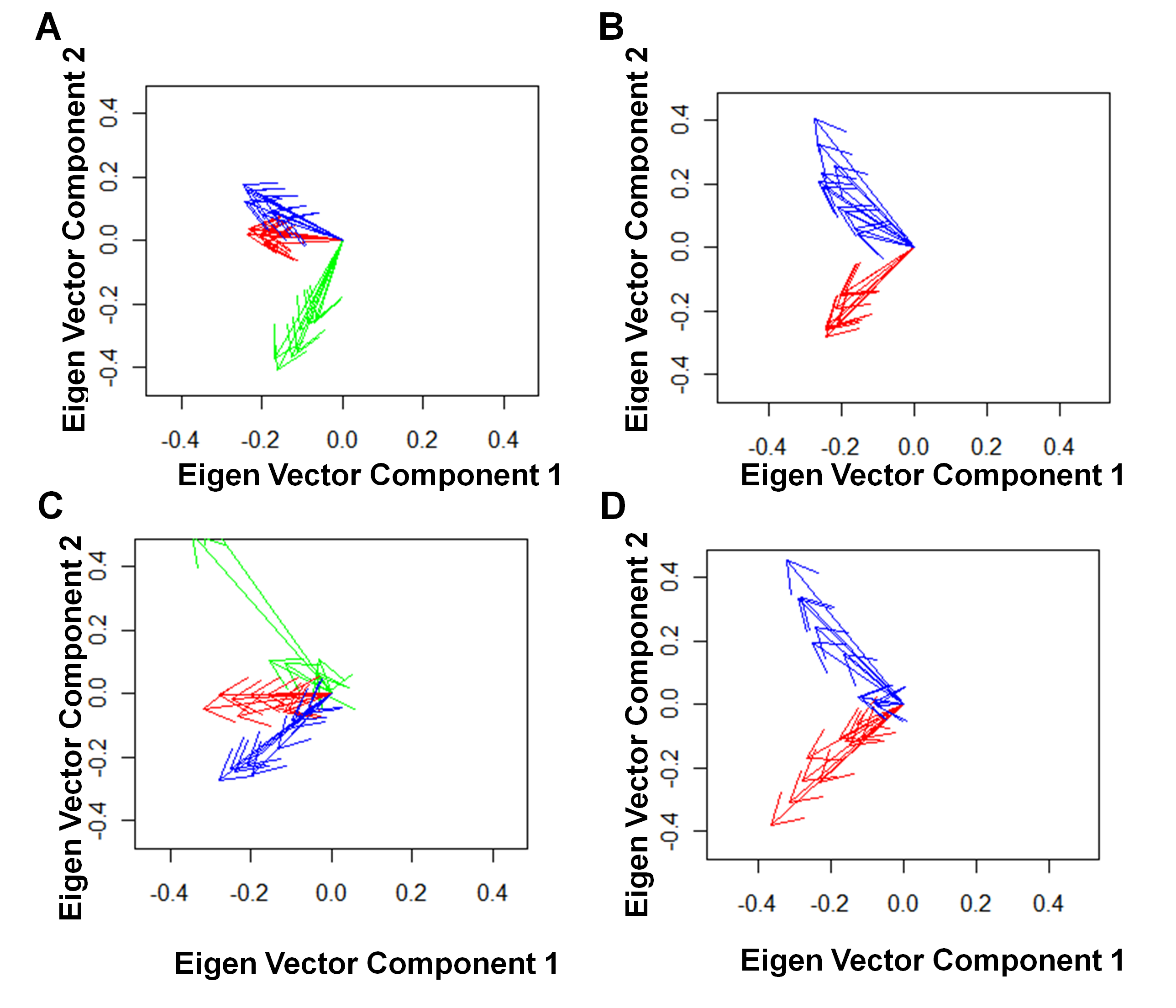

Supplement: Figure S3 — Three groups of joints regardless of different sets of RA patients. Analysis using one of four resampled assessments in one of the two sets of RA patients is shown as a representative. The 1st and 2nd components of eigen vectors of the joint symptoms are plotted, using principal component analysis of the 28 joint involvement for tenderness (A) and swelling (C) or using that of the 20 joint involvement other than large and wrist joints for tenderness (B) and swelling (D). Green: large and wrist joints. Red: MCP joints. Blue: PIP joints. (TIF) [file pone.0059341.s003.tif]

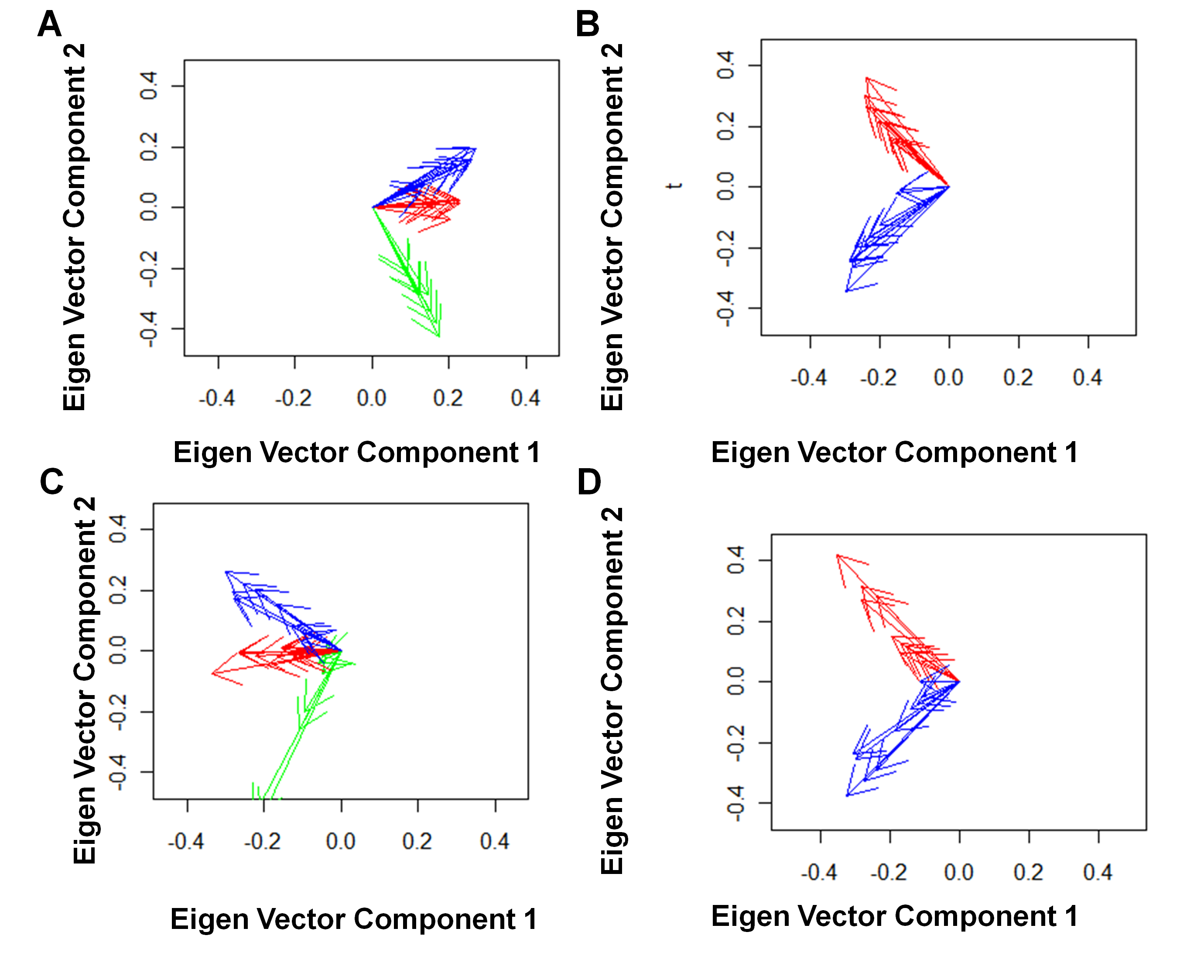

Supplement: Figure S4 — Three groups of joints regardless of different evaluators. Analysis using one of five resampled assessments by one of the two groups of medical doctors is shown as a representative. The 1st and 2nd components of eigen vectors of the joint symptoms are plotted, using principal component analysis of the 28 joint involvement for tenderness (A) and swelling (C) or using that of the 20 joint involvement other than large and wrist joints for tenderness (B) and swelling (D). Green: large and wrist joints. Red: MCP joints. Blue: PIP joints. (TIF) [file pone.0059341.s004.tif]

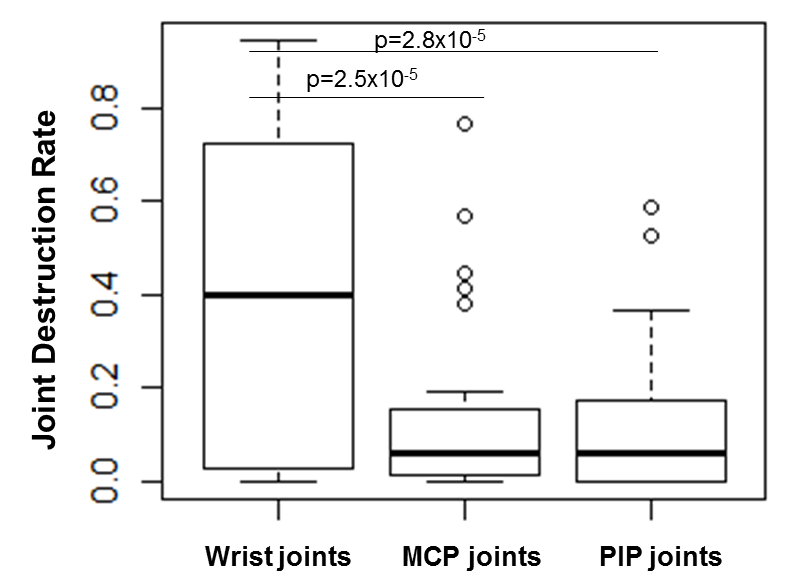

Supplement: Figure S5 — Dominant destruction of large and wrist joints in the sixth subgroup of patients with RA. Box plots indicating the joint destruction rates in the three joint groups in subjects belonging to the sixth subgroup. (TIF) [file pone.0059341.s005.tif]

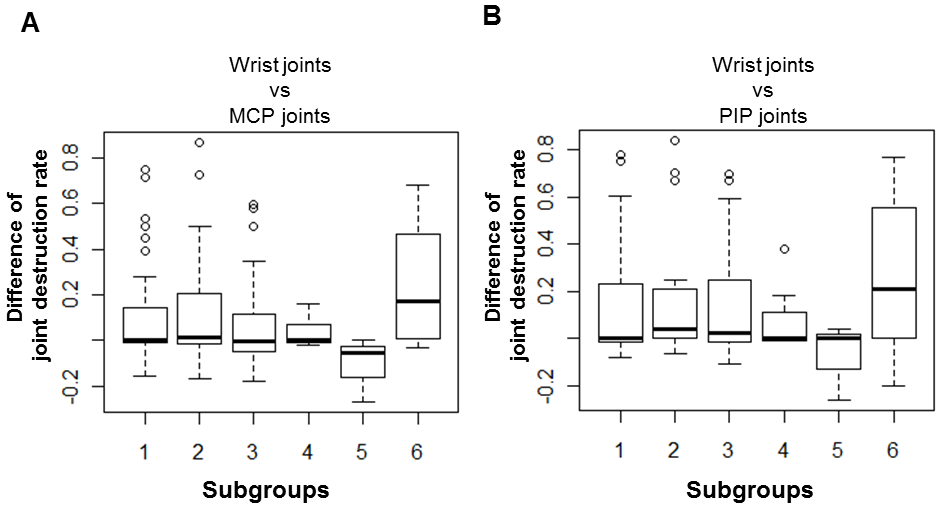

Supplement: Figure S6 — Destruction of large and wrist joints among the six subgroups of RA. Differences in destruction rates were plotted for each subject in the six subgroups. The difference was defined as: A) destruction rate of group of large and wrist joints – destruction rate of MCP joints and B) destruction rate of group of large and wrist joints – destruction rate of PIP joints. (TIF) [file pone.0059341.s006.tif]
