# Supplementary material for: Three Groups in the 28 Joints for Rheumatoid Arthritis Synovitis – Analysis Using More than 17,000 Assessments in the KURAMA Database
Source: PLoS One. 2013 Mar 12;8(3):e59341. doi: 10.1371/journal.pone.0059341 (PMC3595245; doi:10.1371/journal.pone.0059341)
Supplement: Table S1 — Rate of joint involvement for 28 joints in RA. (DOC) [file pone.0059341.s007.doc]

|  | Swelling | | Tenderness | |
| --- | --- | --- | --- | --- |
|  | right | left | right | left |
| shoulder | 0.5 | 1.0 | 5.4 | 5.0 |
| elbow | 6.8 | 5.6 | 5.9 | 5.4 |
| wrist | 17.6 | 17.6 | 14.3 | 12.8 |
| knee | 8.8 | 9.0 | 9.5 | 9.1 |
| 1MP | 3.5 | 2.2 | 5.9 | 3.3 |
| 2MP | 11.0 | 7.3 | 6 | 4.2 |
| 3MP | 9.8 | 6.3 | 4.4 | 3.5 |
| 4MP | 3.9 | 3.3 | 2.3 | 2.9 |
| 5MP | 2.4 | 2.4 | 1.6 | 2.0 |
| 1PIP | 2.7 | 1.9 | 1.9 | 1.6 |
| 2PIP | 5.3 | 5.6 | 7.2 | 4.9 |
| 3PIP | 10.5 | 9.0 | 8.6 | 7.3 |
| 4PIP | 4.9 | 3.8 | 6.5 | 4.2 |
| 5PIP | 1.5 | 1.5 | 2.9 | 3.1 |
