# Supplementary material for: Three Groups in the 28 Joints for Rheumatoid Arthritis Synovitis – Analysis Using More than 17,000 Assessments in the KURAMA Database
Source: PLoS One. 2013 Mar 12;8(3):e59341. doi: 10.1371/journal.pone.0059341 (PMC3595245; doi:10.1371/journal.pone.0059341)
Supplement: Table S2 — Right-dominant joint destruction in RA. Patients who showed unilateral higher or lower scores in each element were analyzed. (DOC) [file pone.0059341.s008.doc]

| Joints | Joint destruction | Rate of patients with right-dominant joint destruction | p-value |
| --- | --- | --- | --- |
| PIP joints | Erosion | 81/135 | 0.025 |
| PIP joints | Narrowing | 34/64 | 0.71 |
| MCP joints | Erosion | 85/133 | 0.0017 |
| MCP joints | Narrowing | 69/108 | 0.0050 |
| Wrist joints | Erosion | 73/136 | 0.44 |
| Wrist joints | Narrowing | 54/111 | 0.85 |
