# Supplementary material for: Three Groups in the 28 Joints for Rheumatoid Arthritis Synovitis – Analysis Using More than 17,000 Assessments in the KURAMA Database
Source: PLoS One. 2013 Mar 12;8(3):e59341. doi: 10.1371/journal.pone.0059341 (PMC3595245; doi:10.1371/journal.pone.0059341)
Supplement: Table S3 — Mean affected rates of the three joint groups in the six subgroups of patients with RA. (DOC) [file pone.0059341.s009.doc]

| subgroups | Large and wrist joints | MCP joints | PIP joints |
| --- | --- | --- | --- |
| 1 | 0 | 0 | 0 |
| 2 | 0.20 | 0.019 | 0.017 |
| 3 | 0.072 | 0.28 | 0.13 |
| 4 | 0.20 | 0.091 | 0.60 |
| 5 | 0.74 | 1.02 | 0.93 |
| 6 | 0.60 | 0.081 | 0.070 |
